# Studies in the Medical Botany of Southern Illinois

**Published:** 1881-10

**Authors:** J. M. G. Carter


### ARTICLE V.

### STUDIES IN THE MEDICAL BOTANY OF SOUTHERN ILLINOIS.

By J. M. G. CARTER, A.M., M.D.

The study of botany is exceedingly interesting, not only on account of the pleasure derived from considering the endless varieties of plant, flower and fruit, but on account of the healthful exercise and growth of mind connected with its observations, investigations and classifications. The study becomes doubly interesting when in addition to the foregoing we take into consideration, as the physician does, the remedial value of the active principles of plants in disease. Whatever is life-saving or health-giving has a peculiar charm about it, and when we consider that the vegetable kingdom furnishes us most of these desirable agents, we have probably the strongest incentive to its study which could draw the mind to the consideration and investigation of any science known to man. Every physician ought to be acquainted with the science of botany so far as it pertains to medicine, and especially the medical botany of his own neighborhood. Not unfrequently he is called to a case (particularly is this so in country practice), where a medicine is required which he does not happen to have with him, but which may be growing in the woods near by. An acquaintance with this branch of medicine at such a time as this can easily be appreciated.

I do not pretend to say that there is a relation between the diseases of a country and its flora, nor venture an opinion as to what that relation, if there is one, may be; but I believe it is an established fact, that where a particular disease seems to be indigenous, *there* is found in the vegetable kingdom a remedy for its cure, and very frequently the best remedy. In this place I might refer to malaria in all its forms, so tenacious in this part of the State, to relieve which we have wahoo, dogwood, mandrake, etc. The same disease prevails in South America, and no remedy has a wider reputation than cinchona, of the same country.

A large proportion of all the medicinal plants indigenous to the United States can be found in Southern Illinois; but those that are used especially in the treatment of diseases of this climate are most abundant.

In this paper I can only refer to the families of plants and some of the more important individuals; those not supposed to be well known, and those having some interesting remedial application in domestic practice among the people of this portion of the State. For descriptions of these plants, I must refer the reader to Gray's Manual, or any other good manual of the botany of Illinois.

The crowfoot family (Ranunculaceæ), the order to which aconite and coptis belong, is represented in Southern Illinois by a score or two of medicinal plants, the most important of which are Hydrastis Canadensis and Cimicifuga Racemosa. Yellow puccoon root is frequently used in decoction or infusion for "chills," jaundice and debility, with excellent effect. It is also used with benefit as a mouth wash in various forms of stomatitis. I use the powdered root with chlorate of potassa or alum, in ulcerated sore mouth, and I have often found the combination to be superior to the potash alone. The people not uncommonly use an infusion of the root as a wash for ulcers. Cohosh, or blacksnake root, is often used in rheumatism, dropsy and fevers.

The magnolia family (Magnoliaceæ) has but one representative so far as I know. This is the Liriodendron Tulipifera. It is a magnificent tree, generally called poplar. The bark of any part, especially the root, may be used in infusion or powder as a tonic, and in malarial fevers, more particularly intermittent. The ordinary strength of the infusion is ℥j to Oj of water. The powder may be given in doses of 30 to 60 grains.

Podophyllum Peltatum is the only plant of the barberry family (Berberidaceæ) which deserves mention in this paper. This plant, known as the May apple, or mandrake, is largely used in this portion of the State in bilious fevers (remittent and intermittent); it is sometimes combined with calomel and sometimes given alone, or in combination with other vegetable cathartics. In these fevers, when it is necessary to keep up cathartic action for several days, I frequently give two or three full doses of calomel, and follow with podophyllum and blue mass. In splenic dropsy mandrake in connection with tonic doses of quinine is a most valuable remedy.

Poppy family (Papaveraceæ). I am not aware of any indigenous variety of the poppy in Southern Illinois, but the Papaver Somniferum is occasionally cultivated and used in domestic practice, in infusion or decoction, as an anodyne to sores, and sometimes internally. It does not seem to be as strong as the eastern or continental plant; perhaps because not so much care is bestowed upon its cultivation here as in the old world. I know of no experiments with the poppy produced here to determine its value; but presume its value is equivalent to that of any other portion of the United States. Opium, in its various forms, is very abundantly employed in both general and domestic practice, in the malarial diseases of this section. However, there is quite a difference of opinion in regard to the relative value of the various preparations. Some use the pulverized opium, many Dover's powders, and many more morphine. The great drawback to the employment of any of these through the stomach, is their constipating effect, as it is considered by nearly all physicians in this country that the bowels should be opened immediately in bilious fevers. To avoid the constipating tendency of opium, some prescribe svapnia, others use morphine hypodermically. My experience with the hypodermic injection of morphia has not been extensive enough to make me feel safe in making a positive statement that so used it does not constipate; still I have thought that I could see that it was not likely to do so, and many physicians are very confident in their assertions that used in this way, morphia does not affect the bowels.

Sanguinaria Canadensis, another plant of the poppy family, is very abundant in this part of the State, being known by the name of *red puccoon root.* Blood-root is used in general and domestic practice as an alterative and tonic chiefly, in amenorrhœa and inactive liver, in cases of chronic malaria, etc. It may be used in combination with Podophyllum Peltatum in splenic dropsy. It is used by the laity principally as "bitters," the dried root in some form of spirituous liquor—usually whisky. Occasionally it is so used in combination with Hydrastis Canadensis. It must be used in small doses, and continued for considerable time. I had a case of acute jaundice last January, due to obstruction of the gall duct—an effect of malaria. The case became chronic, and I used sanguinaria for two months, with ultimate recovery.

The mustard family (Cruciferæ) is another order with a large representation in our section, but I shall mention only three, the others not being of any great importance. Nasturtium Armoracia (horse-radish), Sinapis Nigra (black mustard), and Sinapis Alba (white mustard), are very important plants, and the first two espcially are used in domestic practice as emetics and counter-irritants. Not unfrequently, when I have not been able to secure mustard, I have had horse-radish scraped, and used as a poultice to produce counter-irritation in bilious pneumonia, or "winter fever." The results have been good, and I have no doubt that it might be safely used as a substitute for mustard in any case where mustard is indicated, provided care be taken. The mustards (black and white) do not seem to be indigenous, but were first brought from Europe, and have now escaped from gardens. Horse-radish is native in this State. Treacle mustard (Erysimum), and hedge mustard (Sisymbrium) may be used as substitutes.

Besides prickly-ash (Zanthoxylum), the rue family (Rutaceæ) contains wafer-ash, or hop-tree (Ptelea Trifoliata). Wafer-ash is an excellent tonic to the stomach and intestines, especially in chronic malarial and typhoid cases. Combined with pepsin, it is greatly beneficial in dyspepsia. I have used it with good results, thus combined, in cases where sometimes "heartburn" and sometimes distressing pain is felt after eating. I have often given it four or five hours after a meal, when the stomach is disturbed and painful, and have seldom known it to fail to give immediate relief. An excellent preparation is manufactured by Chapman & Green, of Chicago.

The remedy may be used in infusion, or better, in tincture prepared, according to Dr. Potter, by the following formula:

℞. Hoptree bark........................℥ vi.
Ginger...............................℥ ss.
Whisky...............................O iv.

M. Sig. Tablespoonful thrice daily.

The Cashew family (Anacardiaceæ) also contains some plants of considerable remedial value. In a country where so much calomel is used as in this, and where at least half the people have been salivated, it is of great service to have a remedy specially adapted to cure such cases of sore mouth. Sumach (Rhus Glabrum) has a high reputation in this malady. It has also been recommended as a cooling drink in febrile complaints. An infusion of the berries is used. It is somewhat astringent.

Poison ivy, or poison oak (Rhus Toxicodendron), concerning which so much has been said and written within the past few years, belongs to this family, and is very abundant in Southern Illinois. The poison vine (R. Radicans) is also abundant. The last two plants, and some others of the same family, are not used, so far as I know, in domestic practice, and are only feared by the people for their poisonous properties.

The principal representative of the staff-tree family is wahoo (Euonymus), or burning-bush, sometimes called Indian Arrow. The variety I speak of is Euonymus Atropurpureus. I have already mentioned this as an important remedy in malaria.

This plant, or shrub (Nat. Order Celastraceæ), is found in low grounds and swamps. The bark is the part used, an infusion of which is often taken as a tonic in chills and fever. It has been used with success in dropsies. In its cathartic action it somewhat resembles rhubarb. It is a diuretic, and supposed to be an alterative, acting especially on the liver. It is used in infusion in splenic dropsy.

Ths large rose family (Rosaceæ) is well represented in our woods. The wild cherry (Prunus Virginiana, P. Scrotina) belongs to this family, and is abundantly used as a tonic with iron, quinine and wahoo. Its tonic value is seen in general debility, malaria, and hectic fever of consumption. To this family belong also strawberry (Fragaria Illinoiensis), raspberry (Rubus Odoratus, R. Strigosus, R. Occidentalis), blackberry (Rubus Villosus), and dewberry (Rubus Canadensis). The root of all these may be used similarly to the blackberry of the U. S. Dispensatory.

The saxifrage family (Saxifragaceæ), in addition to several other medicinal plants, is represented in alum-root (Heuchera Americana). This root is used where other astringents would be indicated, and at one time had quite a reputation as a cancer medicine. It may be used in powder, decoction or infusion.

Passing by Hamamelis and Liquidambar, of witch-hazel family, Œnothera, of primrose family, all of which are valuable remedial agents, I mention the parsley family (Umbelliferæ), chiefly because it contains Conium Maculatum, a most excellent substitute for opium when it is desired to avoid constipation. This plant is said to grow in Southern Illinois; however, I have not seen it. Deucus Carota, common carrot, belongs to the same family. An infusion of the root is an excellent nephritic remedy in chronic cases. It is used in dropsy. Water hemlock (Cicuta), parsnip (Pastinacea), and other indigenous medicinal plants belong to the same family.

To the ginseng family (Araliaceæ) belong false sarsaparilla (Aralia Nudicaulis) and ginseng (Panax Quinquefolium). The root of the latter is used in whisky, by some people in this part of the State, as a general tonic. This plant is valued very highly by the Chinese, as a remedy in all diseases.

The dogwood family (Cornaceæ) is another family useful in medicine. Dogwood (Cornus Florida) is an excellent substitute for quinine. It is not unfrequently used as a tonic, in the form of a tea or bitters made of the bark. When quinine has failed in intermittents, dogwood often will effect a cure. Other varieties of dogwood (C. Cincinata, C. Sericea, C. Asperifolia), may be used as substitutes for Cornus Florida.

Only fifteen of the thirty-one orders, or families, of polypetalous exogenous plants, found in Southern Illinois, that contain medicinal plants, have been mentioned; and these thirty-one orders do not contain forty per cent. of the medicinal plants indigenous to this portion of the State. The fifteen orders mentioned contain the most important remedies of this botanical division. The remaining botanical divisions are left for future study.

This outline of the medicinal plants in only fifteen families is sufficient to show that our State is rich in medicinal agents. One hundred plants might have been given, instead of less than forty; but perhaps the list has been sufficiently extended.

